# How Do Consumers Trust and Accept AI Agents? An Extended Theoretical Framework and Empirical Evidence

**DOI:** 10.3390/bs15030337

**Published:** 2025-03-10

**Authors:** Xue Zhao, Weitao You, Ziqing Zheng, Shuhui Shi, Yinyu Lu, Lingyun Sun

**Affiliations:** College of Computer Science and Technology, Zhejiang University, Hangzhou 310058, China

**Keywords:** AI agent, heuristic–systematic model, trust, acceptance, structural equation model

## Abstract

With the rapid development of generative artificial intelligence (AI), AI agents are evolving into “intelligent partners” integrated into various consumer scenarios, posing new challenges to conventional consumer decision-making processes and perceptions. However, the mechanisms through which consumers develop trust and adopt AI agents in common scenarios remain unclear. Therefore, this article develops a framework based on the heuristic–systematic model to explain the behavioral decision-making mechanisms of future consumers. This model is validated through PLS-SEM with data from 632 participants in China. The results show that trust can link individuals’ dual decision paths to further drive user behavior. Additionally, we identify the key drivers of consumer behavior from two dimensions. These findings provide practical guidance for businesses and policymakers to optimize the design and development of AI agents and promote the widespread acceptance and adoption of AI technologies.

## 1. Introduction

In recent years, the rise in generative artificial intelligence (AI) and large language models (LLMs) has marked a revolutionary advance in the field of AI (Hadi et al., 2023). AI agents, the entities that can replace humans to autonomously perform specific tasks (Wiesinger et al., 2024), are evolving from traditional single-task execution tools to intelligent partners capable of independently executing complex decisions and behaviors. Technological breakthroughs have equipped AI agents with stronger language understanding, generation, and adaptive capabilities (Xi et al., 2023). For example, current AI agents can not only replace humans in making certain levels of cognitive decisions (Badghish et al., 2024; Ali et al., 2024), but also partially replace human embodied behaviors (Fu et al., 2024). Shortly, AI agents may fundamentally change the way humans live.

The aforementioned disruptive changes in technology have already profoundly reshaped consumer cognition and behavior. In particular, AI agents, which exhibit far more intelligent and humanized characteristics (Hayawi & Shahriar, 2024), not only deepen the complexity and diversity of consumer cognition, but also fundamentally change consumer trust mechanisms. For example, compared to algorithms, people seem to trust human judgment more, leading to hesitation toward AI (Ananthan et al., 2023). This lack of trust will inevitably become a major barrier to the widespread adoption of AI agents in the near future. Therefore, with the proliferation of AI agents in various forms in different scenarios (McKinsey Global Institute, 2023; World Economic Forum, 2023; Ipsos MORI, 2024), a crucial question arises: How do consumers build trust and acceptance of AI agents as a comprehensive category rather than a specific tool?

Previous research on AI trust has provided valuable insights. Nevertheless, some limitations warrant further exploration. First, the conclusions of the research do not have universal value due to the limitations of the research scenario. The existing results are mostly focused on specific domains, such as healthcare (Zhang et al., 2023), e-commerce chatbots (Chakraborty et al., 2024), and travel planning (Shi et al., 2021). The design premise of these studies stems from the single-task attribute of traditional AI, which limits research conclusions to expert barriers and makes it difficult to explain the common consumers’ trust laws when faced with generalized agents. This dilemma is particularly pronounced in the current context of AI breaking domain boundaries and evolving into a full-scenario intelligent ecosystem. In other words, AI agents are entering every aspect of our lives, and there is an urgent need to establish a cross-domain and common trust framework. Second, from a theoretical perspective, path dependence hinders the unpacking of the micro-foundations of trust itself. Although existing studies have analyzed the correlation between trust and acceptance through traditional paradigms, such as the Technology Acceptance Model (TAM) (Choung et al., 2023) and the Unified Theory of Acceptance and Use of Technology (UTUAT) (Roh et al., 2023; Abadie et al., 2024), they cannot further identify the underlying cognition of trust formation. Later research discussed the components of trust, such as dividing it into emotional trust (a form of trust based on a user’s emotional resonance and subjective feelings toward a target object) and cognitive trust (a form of trust based on a user’s rational evaluation of the target object) (Kyung & Kwon, 2022). Although this provides great inspiration, this classification seems unable to capture the comprehensiveness of trust and deserves further expansion and discussion. Third, the single dimension of antecedents separates the systematic construction of trust. Existing research often focuses on only one type of attribute, resulting in a disconnect between the combined effects of technical attributes and user characteristics on trust. For example, some studies consider only technical attributes (J. Kim & Im, 2023), while others consider only user characteristics (Huo et al., 2022). This leads to research conclusions that cannot objectively reflect real-life situations and cannot comprehensively reveal the driving force of user trust.

In response to these research limitations, this article proposes an extended theoretical framework to explain consumers’ behavioral responses to AI agents, aiming to understand the construction process of trust and acceptance from the perspective of behavioral decision-making. Compared to previous studies, our research has the following novelties. First, we explore the public’s perspective on AI agents as a broad category rather than a specific application or tool through the design of survey scenarios. By doing so, it achieves a transition from the specialized domain to the general domain and constructs a trust framework applicable to AI agents in a wide range of scenarios. Second, this study constructs a three-stage dynamic model of cue–trust–behavior based on the heuristic–systematic model (HSM) (Chaiken, 1980), which is a dual-path decision theory. Based on the existing trust classification (emotional trust and cognitive trust), the concept of overall trust was expanded in this paper, making the trust mechanism more stable. Third, regarding the antecedents of trust, we consider both user characteristics and technical features in each decision path to reflect the true driving process of trust more comprehensively and objectively. Fourth, based on the current technological development background, we discuss users’ ethical expectations of AI agents and provide demand-oriented practical guidance for the design and development. This theoretical framework was validated with empirical data from 632 participants, and all hypotheses were supported. The results reveal the cogwheel effect of trust, which means that trust can transform static basic characteristics into positive behavioral intentions toward AI agents. This study promotes research progress on AI trust and acceptance and lays a theoretical foundation for building a human-centered AI governance system. At the same time, the findings provide a multidimensional optimization path of “emotion–cognition–ethics” for the design and development of AI agents, establish a step-by-step strategy for trust cultivation, and enhance the acceptance of human-centered AI solutions.

## 2. Theoretical Background

AI agents have been the subject of academic research since the mid-to-late 1980s (Maes, 1990; Nilsson, 1992; Müller & Pischel, 1994). AI agents are defined as entities that can autonomously perform specific tasks on behalf of humans, attempting to achieve goals by observing the world and using its tools (Wiesinger et al., 2024). Our study follows this definition and focuses on consumers’ perceptions of this comprehensive concept. Early AI agents were mainly used to perform pre-programmed specific tasks, such as automated operations or rule-based decision support (L. Xu et al., 2021; Sànchez-Marrè, 2022). The design of such agents is based on fixed rules and limited interactions, and they lack flexible learning capabilities (Kadhim et al., 2013), making them more like tools. However, in recent years, with breakthroughs in LLMs and generative AI, current AI agents have made significant progress in terms of intelligence and autonomy. The current AI agents are capable of perceiving environmental information, making autonomous decisions, and executing actions to gradually achieve specific complex goals (Xi et al., 2023). Compared to traditional agents, they exhibit more flexible, diverse, and human-like characteristics and are able to perform complex tasks independently. For example, with ChatGPT 4.0 (https://chatgpt.com/ (accessed on 28 February 2025)), users only need to enter simple requests, and it can proactively call multiple tools to progressively complete tasks to meet users’ needs. These changes imply that AI agents are gradually evolving into independent individuals capable of decision-making and execution, which may have a disruptive impact on consumer perception. Therefore, in this section, we propose a theoretical framework to explain consumers’ cognitive and psychological responses to future AI agents.

### 2.1. Trust Model Based on HSM

The heuristic–systematic model (HSM) is one of the most popular models rooted in the dual-process theory, which was first proposed by psychologist Shelly Chaiken (Chaiken, 1980). This model proposes two modes of information processing modes: heuristic processing (also known as the H-path) and systematic processing (also known as the S-path) (Chaiken, 1980). Heuristic processing refers to information processors who use non-content and situational cues to evaluate information and reach evaluative conclusions with minimal cognitive effort. In other words, evaluators may rely on more readily available information (such as personal experience) to make decisions. It is therefore a fast, simple, and cognitively resource-efficient processing method. In contrast, systematic processing is a more thoughtful and meticulous approach to processing. It involves individuals conducting detailed analyses and examinations of the content features of information, forming reasonable cognitions and logic, and then making final decisions. In summary, the HSM mainly assumes that individuals adopt these two modes to process external information and use them as antecedents for attitude formation and behavioral responses (Ryu & Kim, 2015).

**Figure 1:**
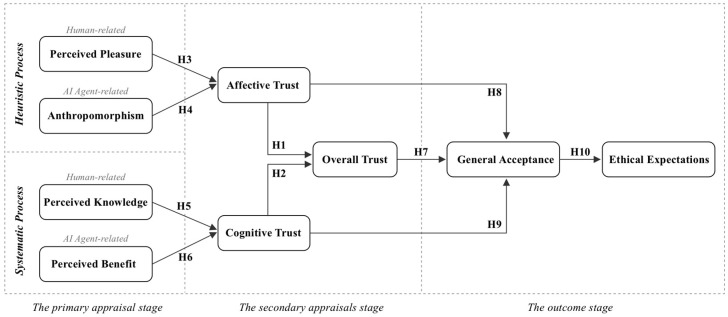
Research model and hypotheses.

Some studies have applied the HSM in the field of AI to understand the interaction patterns between users and AI. The existing works have demonstrated the empirical effectiveness of the HSM in backgrounds such as travel plan recommendation systems (Shi et al., 2021), news recommendations (Shin, 2021), and health chatbots (Y. L. Liu et al., 2022). These studies suggest that both heuristic and systematic processing play a role in evaluating AI output, and ultimately jointly drive user behavior. However, there are still some gaps that need to be explored further. On the one hand, existing research assumes that AI operates within predefined roles, which cannot represent users’ decision-making mechanisms toward AI agents in general scenarios. On the other hand, these studies have overlooked the multidimensionality of cues in different pathways (H-path and S-path). For example, these studies typically only considered the cues related to AI, such as explainability and performance effectiveness (Shi et al., 2021; Shin, 2021), while ignoring the cues related to the user. Therefore, inspired by these shortcomings, this article proposes a comprehensive theoretical framework based on the HSM to explain users’ behavioral responses and decision-making mechanisms toward AI agents (as a comprehensive concept), as shown in Figure 1. This model divides the process of trust establishment into three key stages: the dual-path decision-making process, the trust-linking mechanism, and the behavior-driven process. In the first stage, we propose that trust decisions are driven by dual information processing paths (H-path and S-path) while considering the multidimensionality of cues (human-related and AI agent-related) in each path. Finally, four representative factors are included as antecedents of trust (perceived pleasure, anthropomorphism, knowledge, and perceived benefit). In the second stage, trust is divided into emotional trust and cognitive trust, which ultimately integrate to form overall trust. The third stage is trust driven behavioral response, including acceptance and ethical expectations. The following sections will introduce these variables one by one and propose hypotheses about the relationships between these variables through the literature review.

### 2.2. Components of Trust

Trust, the general willingness to trust others (Mayer et al., 1995), has evolved into distinct dimensions in human–AI contexts. According to previous views, trust includes cognitive trust and emotional trust (Kyung & Kwon, 2022; J.-g. Lee & Lee, 2022). Cognitive trust refers to the cognition formed by users’ rational reasoning and evaluation of the evaluated object, which is based on objective results (Lewis & Weigert, 1985). In other words, users will establish cognitive trust based on rational cognition and the evaluation of the potential benefits of technology (Komiak & Benbasat, 2006). Emotional trust refers to the user’s attitude toward the evaluated object, which is formed by irrational factors (McAllister, 1995; Lahno, 2020) and is usually based on the user’s emotions and feelings. As previously suggested by research, it is necessary to simultaneously examine different types of user trust in the context of AI (Glikson & Woolley, 2020). This is because AI technology exhibits characteristics that mimic human intelligence, which may lead users to compare AI agents to humans during interactions, resulting in complex cognitive and emotional factors that cannot be ignored (Gursoy et al., 2019). Given the complexity and multidimensionality of trust, we believe that even emotional trust and cognitive trust cannot fully encompass the concept of trust. This paper boldly speculates that affective trust and cognitive trust are merely stage-specific psychological products of the two decision-making pathways, which are ultimately integrated by overall trust, thus representing a comprehensive view of an individual’s trust in AI agents. Therefore, building on existing classifications of trust (cognitive trust and emotional trust), we also consider overall trust, defined as the general tendency to be willing to rely on AI agents in a variety of situations (Mayer et al., 1995; McKnight & Chervany, 2001). It will facilitate a more scientific and thorough explanation of the mechanisms by which consumers develop trust in AI agents. In summary, this paper discusses three types of trust and their interrelationships, including affective trust, cognitive trust, and overall trust. We propose the following hypotheses:

**H1.**  *The user’s affective trust in AI agents will positively influence overall trust.*

**H2.**  *The user’s cognitive trust in AI agents will positively affect overall trust.*

### 2.3. Drivers of Trust

When considering the antecedents of user trust, previous studies have typically considered only a single category of factors. However, in the field of human–AI interaction, the characteristics of both interacting entities serve as cues that influence the process of developing human trust. Based on this, we emphasize and consider the multidimensionality of trust antecedents, which addresses the shortcomings of previous research (Yang & Wibowo, 2022). In other words, we include both human- and agent-related factors in the theoretical framework of both decision paths. It is worth noting that only a limited number of representative variables have been selected in this paper, and more variables will be further expanded in future research. Specifically, in the H-path, we selected perceived pleasure (user characteristic) and anthropomorphism (AI agent characteristic) as independent variables. In the S-path, we selected knowledge (user characteristic) and benefit (AI agent characteristic) as independent variables. Based on previous research and the results of meta-analyses, these four variables are often explored and have been shown to have a significant impact on trust (Duffy, 2017; Cabiddu et al., 2022; Venkatesh, 2000; Kaplan et al., 2023). Therefore, we assume that these variables will continue to be effective within our research framework. The following literature section will introduce each variable in turn.

Perceived pleasure is defined as the pleasurable response elicited by AI agents. Research has shown that users’ emotional responses often influence their trust in a particular technology (Lahno, 2001; Komiak & Benbasat, 2006). This finding is consistent with the perspective of affective heuristic theory, which suggests that emotional feedback related to the target object can provide cues to decision evaluators (Slovic et al., 2002). For example, positive emotions provide a “proceed” feedback signal, thus aiding in the quick judgment of whether to approach the object. In other words, in the process of interacting with AI, the emotion of pleasure will serve as a key cue in the heuristic processing path, helping evaluators to quickly respond positively, such as a higher willingness to accept the technology (Venkatesh, 2000; Tan et al., 2022). Based on these perspectives, we speculate that perceived pleasure during the interaction between users and AI agents can help improve users’ affective trust, assuming the following:

**H3.**  *The user’s perceived pleasure of AI agents will positively influence affective trust.*

Social presence theory asserts that the characteristics of the technology itself influence individual perceptions and points out that technology is perceived as more human-like than as a mere tool (Short et al., 1976). The theory emphasizes that the anthropomorphic characteristics of technology warrant attention, which refers to the degree to which users perceive AI agents as possessing human-like awareness and capabilities (H.-Y. Kim & McGill, 2018). However, there is currently no consensus on the relationship between anthropomorphism and user trust, which may be related to the research context. For example, a previous study showed that when AI is anthropomorphized, users tend to trust and follow it regardless of its actual performance (Castelo et al., 2019). Similar conclusions have been supported in different AI application scenarios, such as chatbots (Cheng et al., 2022) and personal assistants (Chen & Park, 2021; Patrizi et al., 2024). However, a study conducted in the banking and telecommunications industry did not seem to find such a significant relationship (Chi & Hoang Vu, 2023). Subsequently, some scholars further considered the different categories of anthropomorphism and emphasized that these categories would affect the relationship between anthropomorphism and trust (Inie et al., 2024). The research objective of this article is to discuss users’ overall anthropomorphic perception of AI agents (as a comprehensive category). Therefore, we have decided to follow the conclusions of most research. In other words, this article speculates that when humans rely on AI agents for decision-making and task execution, the anthropomorphic features of agents can provide users with positive emotional experiences, thereby helping to cultivate higher levels of emotional trust. Therefore, we propose the following hypothesis:

**H4.**  *Anthropomorphism of AI agents will positively influence affective trust.*

Third, knowledge is one of the core concepts for understanding consumer perceptions of new technologies and innovations (Knight, 2005). Without sufficient knowledge of a given issue, people may resort to “mystical beliefs and irrational fears of the unknown” (Sturgis & Allum, 2004). In addition, this paper defines cognitive trust as whether users are willing to accept factual information or suggestions and take action, and whether they believe that the technology is helpful, capable, or useful (Glikson & Woolley, 2020). It follows that cognitive trust is often directly related to the user’s knowledge and understanding of the technology’s capabilities, a view supported by many studies. For example, a study on autonomous driving found that those with the least knowledge had the worst attitudes toward autonomous driving (Sanbonmatsu et al., 2018). In other words, users construct trust based on their understanding of the new technology, while those who lack knowledge cannot reasonably construct trust. Thus, this paper speculates that perceived knowledge associated with AI agents could help people make rational evaluations and thus build cognitive trust. Therefore, we propose the following hypothesis:

**H5.**  *The user’s perceived knowledge about AI agents will have a positive effect on cognitive trust.*

Fourth, perceived benefits are defined in this paper as the advantages and benefits that users believe will result from the use of AI agents. Evaluating potential benefits is a cognitive process based on a rational analysis that influences users’ opinions about certain issues. Typically, when users strongly perceive the benefits of a technology, they tend to exhibit positive attitudes and behavioral tendencies (Hohenberger et al., 2017; Kohl et al., 2018). In other words, when users believe that AI is useful and helpful, they tend to trust it. For example, a study on AI-driven IRAs found that reflecting the potential benefits of information retrieval can strengthen participants’ cognitive trust and thus improve their attitudes (J. Kim, 2020). Therefore, we believe that if future users can perceive the benefits and advantages that AI agents bring to their lives and work, it will help establish a higher level of cognitive trust, thereby promoting their acceptance and willingness to use AI agents. We propose the following hypothesis:

**H6.**  *The user’s perceived benefit of AI agents will positively influence cognitive trust.*

### 2.4. Outcomes of Trust

As an important consequence of user trust, the intention to accept and use AI has become a hot research topic (Nazir et al., 2023). Previous studies consistently show that there is a significant positive correlation between user trust in AI and its adoption. For example, higher levels of trust stimulate higher behavioral intentions among users when interacting with various AI devices (Choi et al., 2023; Xiong et al., 2023). Moreover, even the most classical TAM needs to further consider the predictive role of trust (K. Liu & Tao, 2022; Vorm & Combs, 2022). These findings can be explained by the commitment–trust theory (Morgan & Hunt, 1994), which emphasizes the positive impact of trust on individuals’ commitment to product relationships. Trust has been considered a generic concept in previous studies, which corresponds to overall trust in this study. We propose the following hypothesis:

**H7.**  *The user’s overall trust in AI agents will have a positive effect on general acceptance.*

Similarly, affective trust and cognitive trust are also important predictors of acceptance. On the one hand, analogous to the affective heuristic theory (Slovic et al., 2002), the process of establishing affective trust is intuitive and unthinking. In other words, affective trust helps users simplify the evaluation process, allowing them to rely on intuition to decide whether to accept and use AI agents (Komiak & Benbasat, 2006). On the other hand, the process of establishing cognitive trust is based on logical reasoning and rational evaluation, the conclusion of which may be more reliable for users. In short, once users establish cognitive trust, it will largely determine their subsequent behavior. Combining the above discussions, we speculate that affective trust represents users’ sense of security and comfort in relying on AI agents (Komiak & Benbasat, 2006), which will become the basic requirement for their acceptance. Cognitive trust reflects the level of one’s confidence in the capabilities and performance of AI agents, and the higher the level, the more likely they are to accept and use the agents. Therefore, we propose the following hypotheses:

**H8.**  *The user’s affective trust in AI agents will positively influence general acceptance.*

**H9.**  *The user’s cognitive trust in AI agents will positively influence general acceptance.*

In recent years, AI ethics issues such as loss of privacy, unfairness, and opacity have emerged (Oseni et al., 2021), raising concerns and fears among people. Extensive discussions and efforts are underway in society at large to address the ethics of AI (John-Mathews, 2022). In this context, uncovering users’ core ethical requirements for AI technology has become a key breakthrough point. Based on this, this study proposes the variable of ethical expectations to measure users’ expectations of explainability, transparency, and ethical norms of AI. Although this variable is not a direct result of trust, it is highly necessary to include it in our theoretical framework. This is because deconstructing the relationship between acceptance and ethical expectations can provide actionable design guidelines for AI developers to create AI agents that meet people’s expectations. We propose the following hypothesis:

**H10.**  *The user’s general acceptance toward AI agents will positively influence ethical expectations.*

## 3. Materials and Methods

### 3.1. Data Collection

To validate the theoretical model proposed in this paper, we conducted an online survey through a professional Internet survey platform called Baidu Intelligent Cloud. This platform is one of the largest online survey networks in China, with 17 million users from a wide range of demographic backgrounds, covering 300 cities in China, which is advantageous for collecting data representative of the Chinese population. Considering the linguistic and cultural backgrounds of all respondents, the online questionnaires were distributed in Chinese to ensure accurate understanding and responses. All participants received informed consent before the survey and were fully informed of the research objectives. The data collected were used for academic purposes only, with strict adherence to confidentiality, voluntary participation, and anonymity. In addition, an attention check question (It is important that you pay attention to this study. Please check ‘strongly agree’) was included in the questionnaire to verify that the participants were paying attention and responding meaningfully. We collected 792 responses during the one-week online survey. We excluded 160 invalid responses that did not pass the attention check, were under the age of 18, or were incomplete. In the end, 632 valid responses were used for further analysis, with 55.4% male and 44.6% female.

### 3.2. Instrument Development

Before launching the formal survey, we conducted a pilot study to evaluate the effectiveness of the questionnaire content. The pilot study was conducted in two phases: qualitative interviews and quantitative surveys. In the first phase, five experts (PhDs and researchers with a focus on user experience) and five non-experts were selected to participate in semi-structured interviews. The interviews focused on a comprehensive evaluation of the questionnaire, including the introductory text, scales, and options. The goal was to assess the clarity, ambiguity, and overall design of these elements. In addition, participants were asked to provide open-ended feedback regarding their suggestions for potential improvements to the questionnaire. Following an analysis of the interview transcripts, the wording of the questionnaire was refined to improve its clarity and comprehensibility. This revised version was then subjected to a second round of testing to determine its reliability and validity. We then conducted an online survey using the modified questionnaire with 50 valid responses and analyzed the data for the measurement model. The results showed that the reliability of the questionnaire was met, indicating that the questionnaire is suitable for large-scale research.

The formal questionnaire mainly consists of three main parts. Firstly, the questionnaire commences with an introductory textual section to help participants better understand the research background, which is presented below:

“As a key step in this survey, please read the following text carefully before you answer the questions.

The definition of AI agent: AI agents are entities based on AI technology, which can be physical (such as a robot) or virtual (such as a software program). They have the ability to perceive information from the environment, make autonomous decisions, and carry out actions to achieve specific goals.

The development trend of AI agents: With the high maturity of the technology, AI agents are accelerating to the ground. In the next five years, they will be widely used in education, healthcare, work, and travel, which can help you handle almost any matter. As Bill Gates said, AI agents will revolutionize human lifestyle in the next few years.

Scenario imagination: Please imagine that in the near future, AI agents will be deeply integrated into your daily life and work as intelligent partners. Please rate your views on AI agents based on a wide range of future scenarios.”

Secondly, measurement items for each construct in the theoretical model were presented in turn. To ensure the accuracy of the research results, it was necessary to scientifically measure the content of the theoretical model and validate the research instruments. The majority of the items were therefore derived from existing mature scales and appropriately modified according to the research topic and interview results. The final analysis of the data confirmed that the reliability and validity of the instruments were satisfactory and that they were effective at measuring the intended constructs. Respondents were instructed to rate affective trust (Komiak & Benbasat, 2006) and cognitive trust (J.-g. Lee & Lee, 2022) separately using 4 items adapted from previous studies. Similarly, perceived pleasure (Shin, 2021; Tan et al., 2022; Hsieh, 2023) and perceived knowledge (Celik, 2023; Jang et al., 2023) each included 3 items, and anthropomorphism (Delgosha & Hajiheydari, 2021; K. Liu & Tao, 2022; Sinha et al., 2020; Wu et al., 2023) and perceived benefit (Bao et al., 2022; Said et al., 2023) each contained 4 items, all of which had to be rated subjectively. Subsequently, respondents rated 5 items to assess their acceptance, which included items related to attitude, acceptance, and behavioral intentions (Bao et al., 2022; Tan et al., 2022; Hsieh, 2023; Jang et al., 2023). And ethical expectation consisted of 8 items (Shin, 2021; Celik, 2023). To avoid bias due to conceptual similarity, overall trust was measured last by 3 items (K. Liu & Tao, 2022; Zhao et al., 2022; Jang et al., 2023). All items were measured using a 7-point Likert scale. The last part of the questionnaire was about demographic information, including age, gender, education, and AI experience. Appendix A lists all the items used for the formal survey.

## 4. Data Analysis and Results

In this study, structural equation modeling (SEM) and partial least squares (PLS) techniques were used for data analysis. SEM is a statistical analysis technique that incorporates both measurement and structural models capable of simultaneously addressing the relationships between latent variables and their indicators (Hair et al., 2021). In addition, PLS is a variance-based structural equation modeling technique (Wold, 1975) that facilitates the statistical evaluation of the hypotheses underlying the research model. PLS is suitable for analyzing complex relationships among multiple independent and dependent variables (Lowry & Gaskin, 2014), and it is superior to the covariance-based SEM (CB-SEM) method as a solution method for models containing construct measures (Y. J. Xu, 2011), making it appropriate for this study. Data analysis procedures mainly included demographic information statistics, model validation, and mediation effect analysis. We followed Hair’s recommendations for model testing, including both measurement and structural models (Hair et al., 2014).

### 4.1. Sample Characteristics

The final dataset retained 632 complete and valid responses. A descriptive statistical analysis was performed on gender, age, education, etc. Table 1 shows the demographic characteristics of the respondents and their previous experience with AI products. The gender distribution was relatively balanced, with 55.4% male and 44.6% female. Regarding the representativeness of the sample, all users were randomly selected through the platform, covering more than 300 cities in China. In addition, about 94% of the respondents had experience using AI-related products or tools, indicating that they represent a potential user group for future AI agents. Therefore, we believe that this sample is highly representative and can be used for further analysis.

### 4.2. Measurement Model

After excluding item EE4, the remaining items were used for a formal analysis. The measurement model test consisted of a sequential evaluation of the reliability, validity, and method bias of all constructs. The results indicated that the measurement properties of the scale met the standards.

In terms of reliability, composite reliability (CR) reflects the internal consistency among indicators of a latent variable, with CR values of all variables above 0.700 indicating reliable results, detailed in Table 2. Similarly, Cronbach’s alpha (α), another common reliability measure, showed that the α coefficients of all variables were above the recommended threshold of 0.700, confirming that these constructs meet reliability standards (Bagozzi & Yi, 1988; Urbach & Ahlemann, 2010). In terms of convergent validity, the average variance extracted (AVE) represents the shared variance between a latent variable and its indicators. The AVE indicated that more than half of the variance observed in the items was explained by their underlying constructs, and values of all variables were above the threshold of 0.500, indicating good convergent validity (Schumacker & Lomax, 2004; Henseler et al., 2009). Factor loadings (FLs), the correlation coefficients between each measure item and the latent variable, greater than 0.700 indicate good convergent validity (Fornell & Larcker, 1981), and all measure items in this study met these criteria. For discriminant validity, two methods of analysis confirmed the good discriminant effects of our scale. According to the Fornell–Larcker criterion (Fornell & Larcker, 1981), discriminant validity is considered good if the square root of the AVE of each variable (diagonal elements in Table 3) is greater than its correlation coefficients with different variables, and the data of this study meet this condition. In addition, cross-loadings (CLs), which reflect the contribution of a measure item to other latent variables, showed that the loadings of each latent variable measure item exceeded its CLs, indicating good discriminant validity.

In addition, we excluded the possibility of measurement bias due to the survey method. The uniform measurement method could lead to a certain tendency among participants to rate, thus introducing measurement bias. Harman’s single-factor test (Podsakoff et al., 2003) was used to calculate the Common Method Variance (CMV). The results showed that the total variance extracted by the single factor was 41.28%, which is well below the expected threshold of 50%, indicating no common method bias in this study. Furthermore, the variance inflation factor (VIF) for the inner model was calculated to test the risk of multicollinearity, with VIF values of all variables below 3.00 (Hair et al., 2019), indicating no risk of multicollinearity in the scale. Overall, the results of the above analyses all confirmed the good reliability and validity of the scale. In other words, the various aspects of the measurement model met the standards, allowing the scale data to be used directly in the subsequent structural model analysis.

### 4.3. Structural Model and Hypothesis Test

**Figure 2:**
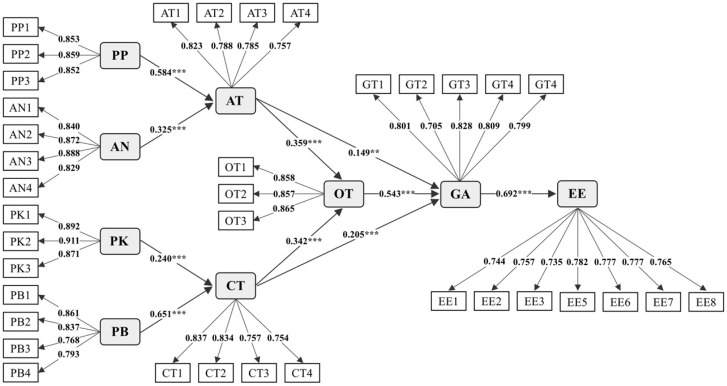
Structural model results. Note: ** *p* < 0.01; *** *p* < 0.001. AN = anthropomorphism, AT = affective trust, CT = cognitive trust, EE = ethical expectation, GA = general acceptance, OT = overall trust, PB = perceived benefit, PK = perceived knowledge, and PP = perceived pleasure.

The first step in the structural model analysis was to test the significance of the relationships between variables. As recommended in a previous study, we conducted 5000 bootstrap samples to test the significance of each path, calculated by bootstrapping. All hypotheses proposed in this article were supported, as shown in Figure 2 and Table 4, based on the calculated path coefficients (*β*) and their significance levels (*p*). As hypothesized in the H-path, perceived pleasure (*β* = 0.584, *p* < 0.001) and anthropomorphism (*β* = 0.325, *p* < 0.001) had a significant positive effect on affective trust. Similarly, in the S-path, perceived knowledge (*β* = 0.240, *p* < 0.001) and perceived benefit (*β* = 0.651, *p* < 0.001) were found to be significant drivers of cognitive trust. Both affective trust (*β* = 0.359, *p* < 0.001) and cognitive trust (*β* = 0.342, *p* < 0.001) had similar influential patterns in facilitating overall trust, and they were statistically significant. In terms of consumer acceptance, overall trust (*β* = 0.543, *p* < 0.001) had the most positive significant impact, followed by cognitive trust (*β* = 0.205, *p* < 0.001) and affective trust (*β* = 0.149, *p* < 0.01). Additionally, this study found that there is a positive correlation between consumer acceptance of AI agents and their future ethical expectation (*β* = 0.692, *p* < 0.001).

The second step in the analysis of the structural model was to evaluate its performance. The evaluation dimensions mainly included the model’s explanatory power, predictive power, and fit of the model, respectively, reflected by the *R*^2^, *Q*^2^, GoF, and SRMR indices. As shown in Table 4, the calculated values of these four indices were all within acceptable ranges, indicating the overall good performance of the model. The results of the PLS algorithm provided *R*^2^ values for four endogenous variables, including overall trust (0.423), cognitive trust (0.575), affective trust (0.650), general acceptance (0.635), and ethical expectations (0.479). *R*^2^ represents the amount of variance in the dependent variables that is explained by the independent variables, and these values exceeded the recommended threshold of 0.200, indicating the good explanatory power of our model (Shi et al., 2021). The *R*^2^ values for cognitive trust, affective trust, and acceptance were higher than those reported in previous studies (Shi et al., 2021), confirming the importance of multidimensional antecedents of trust emphasized in this article. Secondly, *Q*^2^ is related to predictive ability, with values greater than 0 indicating good predictive ability of the model. The blindfolding results showed that *Q*^2^ for affective trust (0.400), cognitive trust (0.360), overall trust (0.309), general acceptance (0.390), and ethical expectation (0.272) all met the standards, suggesting good predictive ability of the model. As a measure of model fit, SRMR ranges from 0 to 1, with values closer to 0 indicating better fit. The SRMR result of this study was 0.063, which is below the recommended value of 0.080 (Hu & Bentler, 1999), indicating a good model fit. Additionally, the GoF value was calculated according to an accepted formula (Tenenhaus et al., 2005) and was 0.613, exceeding the cutoff value for a medium effect size of 0.36, also indicating a good model fit.

To further test the structural relationships in the model, we examined mediation effects, which are detailed in Table 5. Following the rigorous procedure, a step-by-step regression coefficient test method was used to examine mediation effects, and bootstrapping was used to test the significance of these mediation effects. The calculation of 95% confidence intervals indicated mediation effects only when the range did not cross zero. Furthermore, we calculated the variance accounted for (VAF) to determine the strength of the indirect effects (i.e., indirect effects) relative to the total effect (i.e., direct plus indirect effects). VAF values greater than 20% and less than 80% indicated partial mediation (Hair et al., 2016). The results suggest that affective trust mediates the relationships between perceived pleasure–overall trust and anthropomorphism–overall, with VAFs of 31.11% and 79.91%, respectively. In addition, affective trust mediates the relationships between perceived pleasure and general acceptance (VAF = 28.95%) as well as anthropomorphism and general acceptance (VAF = 73.36%). Additionally, cognitive trust was found to have similar mediating effects. Cognitive trust played a significant mediating role in the relationships between knowledge and overall trust (VAF = 35.99%) as well as perceived benefit and overall trust (VAF = 36.15%). It also mediated the indirect influences of knowledge–general acceptance and perceived benefit–general acceptance at VAF values of 37.56% and 52.03%, respectively. Moreover, the overall level of trust played a significant role in mediating the relationships between affective trust and overall acceptance, as well as between cognitive trust and overall acceptance. The mediating effect was strong, with VAFs greater than 50%.

## 5. Discussion and Implications

Trust and acceptance are the fundamental engines for creating human-centered AI (Shin, 2020), as well as the crucial bridge between consumers and future AI agents. Therefore, this study proposes a comprehensive framework to explain consumer trust and acceptance, along with their complex decision-making mechanisms. The results provide robust support for the effectiveness of the proposed model: all of the hypotheses were supported, and the model demonstrated better explanatory power than previous studies (Pelau et al., 2021; J.-g. Lee & Lee, 2022). In addition, this research reveals a crucial mechanism of trust, namely, its ability to integrate the results of dual-path processing, which further influences subsequent user behavior such as acceptance and ethical expectations. Furthermore, this study identifies the key factors that drive different types of trust in different decision pathways, and demonstrates that different types of trust promote user behavioral intentions. In summary, this study provides valuable insights into consumer trust and acceptance in the age of AI agents. It further deepens our understanding of how different types of trust influence consumer behavior and promotes a dynamic perspective on the fundamental characteristics of human and AI agents, trust-building mechanisms, and user acceptance in consumer markets.

### 5.1. Main Findings

Overall, the main contribution of this study is the decoupling of statistical significance and practical differentiation. This not only confirms the statistical significance of each variable, but more importantly, provides empirical evidence for the implementation of differentiation strategies based on effect size analysis. Specifically, on the one hand, the results of this study show that all paths are significant, confirming that the variables in the model have significant explanatory power for the trust and acceptance behavior of AI agents. This phenomenon is consistent with the results of previous research (Venkatesh et al., 2012), which reveals the essential characteristics of the synergistic effect of multidimensional factors on user trust and acceptance in the context of new technologies. In other words, the results of this study also indicate the limitations of traditional single-factor optimization strategies in the era of AI agents. On the other hand, although all paths are statistically significant, the hierarchy of effect sizes reveals the different weights of each variable in practical applications. Specifically, based on the relative size of the path coefficients, we recommend adopting a phased implementation strategy in the practical process. For example, in the early stages of promotion, priority can be given to strengthening the dual driving path of emotional experience design and actual value perception to quickly establish a foundation of user trust. The key findings of this article are discussed in turn below.

Firstly, with the HSM as the theoretical background, the results reveal the cogwheel effect of trust, which means that trust can transform static basic characteristics into positive behavioral intentions toward AI agents. In other words, trust can integrate the processing outcomes of dual pathways for decision-making, thereby further facilitating user behavioral intentions. Simply put, in future scenarios where users interact with AI agents, users will combine the characteristics of themselves and AI agents to process and evaluate trust according to the HSM theory. They establish affective trust by intuitively evaluating heuristic cues, while simultaneously building cognitive trust through the logical analysis of systematic cues. The former provides a simplified evaluation process for quickly judging the credibility of AI agents; the latter relies on deep thinking, combining personal knowledge and the potential value of AI agents for a rational evaluation. Subsequently, the psychological products of these two processing pathways are integrated by overall trust, forming a comprehensive trust conclusion. Ultimately, these different types of trust further promote user acceptance and ethical expectations. This linking mechanism mentioned above is statistically reflected in the mediating effects of affective and cognitive trust. In other words, affective trust and cognitive trust play significant roles in mediating the relationship between the four key drivers and overall trust. Additionally, affective and cognitive trust have a significant indirect effect on acceptance through the bridge of overall trust, in addition to their direct effect on acceptance. In summary, differing from the operational modes pointed out in previous studies (Shin, 2020, 2021), the linking mechanism of trust revealed in this study can serve as an important clue for research on human interactions with AI agents, providing a breakthrough for human-centered AI research.

Second, this article emphasizes the multidimensionality of trust antecedents and identifies the key drivers of affective and cognitive trust based. Specifically, the influencing factors in this model explain affective and cognitive trust better than in previous studies (Shi et al., 2021; J.-g. Lee & Lee, 2022), whose *R*^2^ is much larger. In other words, this conclusion suggests that both human-related and AI agent-related characteristics will serve as crucial cues for individual decision judgments. Only by considering these multidimensional factors can we more fully explain user psychology and behavior. Following this approach, this paper identifies the key factors that influence affective and cognitive trust. For affective trust, it is found that both perceived pleasure (human-related) and anthropomorphism (AI agent-related) significantly positively promote trust, consistent with previous research (J. I. Lee et al., 2023). Concerning the degree of anthropomorphism, simply put, the more similar an AI agent is to a human, the more likely people are to trust the AI agent on affective dimensions. The reason may be that when AI agents exhibit human-like emotions, language, and social behaviors, people are more likely to view them as social partners rather than mere tools, thereby strengthening trust in an affective aspect. It is worth noting, however, that perceived pleasure has a stronger effect on affective trust than anthropomorphism in this study. This finding may follow the affective heuristic theory (Slovic et al., 2007), which means that when people are asked to evaluate complex entities, they first rely on their affective response to make judgments. In other words, when confronted with an AI agent in the future, humans may prefer to rely on their affective reactions and intuition to simplify the decision evaluation process, thereby overcoming the evaluation speed and reliance on anthropomorphism. For cognitive trust, we found that both knowledge and the perceived benefit of AI agents are significant influencing factors. Simply speaking, individuals who believe they have a better understanding of agents are more likely to cognitively trust them. In addition, when consumers perceive greater benefits, they are more likely to develop stronger cognitive trust, which is closely aligned with previous perspectives (J. Kim, 2020). Further findings indicate that perceived benefits have a more significant impact on cognitive trust, about 2.7 times greater than knowledge. This could be explained by the fact that perceived benefits are directly related to the user’s assessment of whether the AI agent can meet their needs. In other words, while some cognitive trust comes from understanding the AI agent, it primarily comes from the expected benefits of using the AI agent. In summary, these findings not only help perfect the understanding of the antecedents of user trust in the AI domain but also provide direct insights for the subsequent design and development of AI agents. For example, in the initial promotion phase, actively demonstrating the potential benefits of AI agents can increase cognitive trust among general consumers. In addition, industry professionals could consider enhancing positive emotional experiences when interacting with AI agents to foster users’ affective trust.

Finally, this paper confirms the importance of considering the multidimensionality of trust, as well as the crucial role of trust in the widespread acceptance of future AI agents. As is well known, trust is often considered a key mechanism to reduce uncertainty and complexity in decision-making processes, especially in the adoption of new technologies. The results of this study indicate that affective trust, cognitive trust, and overall trust are all essential prerequisites for the user acceptance of AI agents. It is worth noting that the approach of considering the multidimensionality of trust provides strong support for its effectiveness in predicting AI agent acceptance, as it explains 59.3% of the variance in AI agent acceptance, surpassing several previous studies (H. Liu et al., 2019; Pelau et al., 2021). This conclusion implies that in the process of implementing AI agents in the future, researchers need to consider the subdivided dimensions of the trust concept to more accurately capture and distinguish the attributions behind user behavior. Furthermore, we find that compared to affective and cognitive trust, overall trust has a stronger facilitating effect on acceptance, which could be explained by the different mechanisms through which different types of trust influence individual behavior. Specifically, cognitive trust is often based on logical reasoning and rational evaluation, while affective trust emphasizes the affective components of trust. In comparison, overall trust represents a more comprehensive, integrated perspective based on the fusion of these two, which can more effectively transfer and promote adoption. In other words, overall trust provides a more comprehensive and stable evaluation basis for the AI agent and thus has a stronger facilitating effect on acceptance. In addition, we revealed an important finding regarding ethical expectations: users who are more inclined to adopt AI agents tend to have stronger expectations and visions for them. For example, they are more likely to hope that the AI agent will adhere to ethical norms of human society, and they expect the AI agent’s processes to remain highly transparent. This may be the result of the high acceptance of user groups’ deep understanding of the AI agent and their high regard for AI ethical issues. In conclusion, the above findings have strong implications. For example, the development of trustworthy AI agents is an inevitable trend, because only by gaining users’ trust can AI agents be widely accepted and truly integrated into human society. In addition, the research and development of AI agents should prioritize ethical considerations to ensure that technological innovation and ethical standards progress in tandem to meet the expectations of potential AI agent user groups.

### 5.2. Theoretical and Practical Implications

Our study makes a significant original contribution and offers several theoretical insights. Most importantly, in response to the demands of earlier phases of AI development, this research re-examines and constructs the consumer cognitive framework for future general AI agents, rather than being limited to specific applications in a particular domain. In addition, this study addresses some of the limitations of previous studies. For example, it adopts HSM to analyze the construction process of user trust, acceptance, and its underlying cognitive mechanisms in the age of AI agents, thus broadening the theoretical and methodological approaches to AI trust and acceptance. In terms of model construction, this research takes into account the multidimensionality of trust antecedents and ethical considerations, thereby enhancing the explanatory power of user trust and behavioral intentions, which is expected to further advance the field of human–machine trust research. Finally, this study reveals that trust is not only a bridge between humans and AI agents but also a dynamic mechanism that transforms static basic characteristics into positive behavioral intentions. This significant discovery provides key insights for understanding consumers’ behavioral patterns in the age of AI agents.

Furthermore, this research has significant practical implications. First of all, compared to prior research, the findings of this research better reflect the perspectives of potential future AI consumers. In addition, this paper clarifies the key antecedents of user trust in AI agents and confirms the mechanism by which trust influences acceptance. This will provide scientifically reliable practical guidance for the design and development of future AI agents, while also urging practitioners to adjust strategies promptly to further promote the development and application of AI agents. For example, improving the level of anthropomorphism of AI agents can be a key strategy to enhance consumer trust, and stimulating positive emotional experiences should be regarded as a foundation for building deep trust relationships. In addition, policymakers in the AI agent industry could consider improving education and communication strategies, such as emphasizing the societal benefits of AI agents and improving the consumer’s understanding of AI agents, to effectively improve consumer trust and acceptance.

### 5.3. Limitations and Future Research

This study has several limitations that can be addressed in future work. The first limitation is that we only focus on consumers’ overall view of agents as a comprehensive category, without further discussion of different agents. Future research can explore in detail the differences in cognitive mechanisms between different types of agents. In addition, we only considered some specific variables as antecedents of trust, which means that future research can investigate more comprehensive variables to enrich this theoretical framework. Furthermore, this study used a cross-sectional survey design to primarily explore non-causal relationships among variables. Future research could employ longitudinal designs and cross-lagged panel models to dynamically examine the evolution of consumer psychological mechanisms, thereby enhancing the validity of causal inferences. Finally, although the definition provided at the beginning of the questionnaire improves measurement consistency, it may also lead to some degree of a potential priming effect, which should be controlled by balanced stimulus variants in future replication experiments.

## 6. Conclusions

Revolutionary advances in AI are bringing humanity into the age of super-agents, which means a complete upheaval in human social patterns, accompanied by significant psychological adaptation challenges. This paper explores the consumer’s trust and behavior mechanisms of future widespread and independent AI agents. Our model provides strategic considerations and a theoretical framework for combining different types of trust with their antecedents and consequences from a decision-making perspective. This research empirically reveals a crucial integrative mechanism in users’ evaluative decision-making: overall trust integrates the results of dual-pathway processing, further influencing the acceptance behavior and ethical expectations of users. In addition, considering the multidimensionality of trust antecedents, this study identifies the key drivers of trust from the human perspective and the AI agent perspective, respectively, and incorporates ethical factors into the model construction. In summary, the long-term goal of AI is to develop human-centered services, and the results of this study are a significant step toward achieving that goal. These insights will guide practitioners and policymakers to take measures to design and implement trustworthy, responsible, and ethically oriented AI agents to meet consumer needs and expectations, thereby further promoting social progress and development.

## Figures and Tables

**Table 1:** Demographic characteristic variables of respondents.

Demographic Characteristics	Variables	N	%
Gender	Female	350	55.4
	Male	282	44.6
Age/year	18–29	350	55.4
	30–39	217	34.3
	40–49	52	8.2
	50–59	12	1.9
	≥60	1	0.2
Education	Middle school and below	14	2.2
	High school	48	7.6
	Junior college	154	24.4
	Undergraduate	317	50.2
	Graduate and above	99	15.7
Experience in using AI-related applications	Presence	593	93.8
	Absence	39	6.2

Note: N = frequency; % = percentage.

**Table 2:** Scales for reliability and validity of measurement models.

Construct	Item	M	SD	FL	α	CR	AVE
Perceived Pleasure (PP)	PP1	5.54	1.312	0.853	0.816	0.891	0.731
	PP2	5.24	1.273	0.859			
	PP3	5.19	1.370	0.852			
Anthropomorphism (AN)	AN1	4.92	1.529	0.840	0.880	0.918	0.736
	AN2	4.35	1.677	0.872			
	AN3	4.50	1.743	0.888			
	AN4	4.84	1.575	0.829			
Perceived Knowledge (PK)	PK1	4.95	1.484	0.892	0.872	0.921	0.795
	PK2	4.66	1.437	0.911			
	PK3	4.31	1.724	0.871			
Perceived Benefit (PB)	PB1	5.82	1.288	0.861	0.831	0.888	0.665
	PB2	5.84	1.177	0.837			
	PB3	5.64	1.302	0.768			
	PB4	5.57	1.216	0.793			
Affective Trust (AT)	AT1	5.18	1.339	0.823	0.797	0.868	0.622
	AT2	5.30	1.271	0.788			
	AT3	5.24	1.346	0.785			
	AT4	4.77	1.560	0.757			
Cognitive Trust (CT)	CT1	5.56	1.293	0.837	0.807	0.874	0.635
	CT2	5.38	1.201	0.834			
	CT3	5.79	1.250	0.757			
	CT4	5.17	1.305	0.754			
Overall Trust (OT)	OT1	5.47	1.149	0.858	0.824	0.895	0.740
	OT2	5.26	1.179	0.857			
	OT3	5.36	1.264	0.865			
General Acceptance (GA)	GA1	5.70	1.233	0.801	0.848	0.892	0.623
	GA2	5.07	1.321	0.705			
	GA3	5.34	1.334	0.828			
	GA4	5.69	1.097	0.809			
	GA5	5.72	1.059	0.799			
Ethical Expectations (EE)	EE1	5.69	1.212	0.744	0.880	0.907	0.582
	EE2	5.66	1.166	0.757			
	EE3	5.56	1.297	0.735			
	EE5	5.78	1.354	0.782			
	EE6	5.59	1.249	0.777			
	EE7	5.72	1.243	0.777			
	EE8	5.76	1.430	0.765			

Note: M = Mean; SD = Standard Deviation; FL = Factor Loading; α = Cronbach’s alpha; CR = Composite Reliability; AVE = Average Variance Extracted.

**Table 3:** Discriminant validity and Correlation Matrix.

Construct	AN	AT	CT	EE	GA	OT	PB	PK	PP
AN	**0.858**								
AT	0.639	**0.788**							
CT	0.472	0.723	**0.797**						
EE	0.315	0.522	0.635	**0.763**					
GA	0.475	0.626	0.640	0.692	**0.789**				
OT	0.445	0.606	0.601	0.569	0.757	**0.860**			
PB	0.328	0.585	0.723	0.728	0.663	0.563	**0.816**		
PK	0.540	0.485	0.435	0.300	0.564	0.550	0.299	**0.892**	
PP	0.539	0.759	0.702	0.572	0.676	0.647	0.682	0.437	**0.855**

Note: Bold-faced diagonal elements are the square roots of AVEs. The off-diagonal elements are the correlations between constructs. AN = anthropomorphism, AT = affective trust, CT = cognitive trust, EE = ethical expectation, GA = general acceptance, OT = overall trust, PB = perceived benefit, PK = perceived knowledge, and PP = perceived pleasure.

**Table 4:** The results of hypothesis testing.

Dependent Variable	Hypothesis	*R* ^2^	*β*	*p* Value	Hypothesis Supported	*Q* ^2^
OT	H1: AT → OT	0.423	0.359 ***	0.000	Yes	0.309
	H2: CT → OT		0.342 ***	0.000	Yes	
AT	H3: PP → AT	0.650	0.584 ***	0.000	Yes	0.400
	H4: AN → AT		0.325 ***	0.000	Yes	
CT	H5: PK → CT	0.575	0.240 ***	0.000	Yes	0.360
	H6: PB → CT		0.651 ***	0.000	Yes	
GA	H7: OT → GA	0.635	0.543 ***	0.000	Yes	0.390
	H8: AT → GA		0.149 **	0.001	Yes	
	H9: CT → GA		0.205 ***	0.000	Yes	
EE	H10: GA → EE	0.479	0.692 ***	0.000	Yes	0.272
GoF = 0.613; SRMR = 0.063						

Note: ** *p* < 0.01; *** *p* < 0.001. AN = anthropomorphism, AT = affective trust, CT = cognitive trust, EE = ethical expectation, GA = general acceptance, OT = overall trust, PB = perceived benefit, PK = perceived knowledge, and PP = perceived pleasure.

**Table 5:** The results of the mediating effect test of trust.

Mediator	Path	CI	VAF (%)	Mediating Role of Trust
AT	PP → GA	[0.1017, 0.2345]	28.95	Partial mediation
	PP → OT	[0.1185, 0.2621]	31.11	Partial mediation
	AN → GA	[0.1870, 0.2875]	73.36	Partial mediation
	AN → OT	[0.2032, 0.3164]	79.91	Partial mediation
CT	PK → GA	[0.1070, 0.1840]	37.56	Partial mediation
	PK → OT	[0.1051, 0.1851]	35.99	Partial mediation
	PB → GA	[0.1522, 0.2951]	52.03	Partial mediation
	PB → OT	[0.2080, 0.3878]	36.15	Partial mediation
OT	AT → GA	[0.2512, 0.3770]	57.18	Partial mediation
	CT → GA	[0.2605, 0.4051]	54.23	Partial mediation

Note: AN = anthropomorphism, AT = affective trust, CT = cognitive trust, GA = general acceptance, OT = overall trust, PB = perceived benefit, PK = perceived knowledge, and PP = perceived pleasure.

## Data Availability

Data will be made available on request.

## Appendix A. The Survey Questionnaire Items

**Construct**	**Scale Items**
Perceived Pleasure (PP)	PP1: Interacting with AI agents will be very fun. PP2: The actual process of using AI agents will be enjoyable. PP3: Using AI agents will be pleasant.
Anthropomorphism (AN)	AN1: AI agents will experience emotions. AN2: AI agents will have consciousness. AN3: AI agents will have minds of their own. AN4: AI agents will have personalities.
Perceived Benefit (PB)	PB1: AI agents will provide convenience for daily life. PB2: AI agents will improve work efficiency and productivity. PB3: AI agents will promote economic development. PB4: AI agents will provide personalized services to better meet individual needs.
Perceived Knowledge (PK)	PK1: I know AI agents very well. PK2: Compared to most people, I have a better understanding of AI agents. PK3: Among the people I know, I can be regarded as an “expert” in the field of AI agents.
Cognitive Trust (CT)	CT1: AI agents will be technologically trustworthy. CT2: The overall ability and performance of AI agents will be reliable. CT3: AI agents will have rich and powerful domain knowledge. CT4: AI agents will make accurate and wise decisions.
Affective Trust (AT)	AT1: AI agents will closely follow and understand my needs in future interactions. AT2: AI agents will provide timely assistance when I need help. AT3: AI agents that help me with tasks will make me feel comfortable. AT4: AI agents will demonstrate understanding and resonance with my emotional state.
Overall Trust (OT)	OT1: AI agents will be reliable. OT2: AI agents will be trustworthy. OT3: Overall, I can trust AI agents in the future.
General Acceptance (GA)	GA1: I will use AI agents in the future. GA2: I will pay for AI agents in the future. GA3: I will recommend AI agents to my family and friends. GA4: Please rate your overall attitude toward future AI agents. GA5: Please indicate your acceptability level of future AI agents.
Ethical Expectation (EE)	EE1: I expect AI agents to provide me with sufficient information to explain their operational principle when performing tasks in the future. EE2: I expect explanations from AI agents to be clear and easy to understand in the future. EE3: I expect AI agents to fully demonstrate their action to me, ensuring transparency in the future. EE4: I expect AI agents to enable me to clearly understand how they make decisions in the future. EE5: I expect AI agents to follow the moral and ethical standards of human society in the future. EE6: I expect AI agents to be fair, such as to ensure that every user receives support in the future. EE7: I expect AI agents to be inclusive, such as avoiding gender discrimination in the future. EE8: I expect AI agents to effectively protect my personal privacy and data security in the future.
Note: All items were adapted from established studies and refined based on the findings from the pre-experiment.	
